# Prior-guided feature fusion for tongue image-based gastrointestinal disease auxiliary diagnosis

**DOI:** 10.3389/fphys.2026.1811717

**Published:** 2026-04-29

**Authors:** Ying Zhu, Zili Zhou, Yuchao Cheng, Jiayu Zhang, Hong Mao, Ling He, Jing Zhang

**Affiliations:** 1 School of Biomedical Engineering, Sichuan University, Chengdu, China; 2 Department of Gastroenterology, Sichuan Provincial Second Traditional Chinese Medicine Hospital, Chengdu, China; 3 School of Business, Singapore University of Social Sciences, Singapore, Singapore; 4 Department of Anorectal Surgery, Sichuan Provincial Second Traditional Chinese Medicine Hospital, Chengdu, China

**Keywords:** channel attention, computer-aided diagnosis, gastrointestinal diseases, gated feature fusion, tongue image analysis

## Abstract

**Introduction:**

Gastrointestinal diseases (GIDs) are a major global health burden, but traditional endoscopic screening faces limitations due to its invasiveness. Tongue image analysis offers a non‑invasive alternative but is subjective, relying on clinical experience.

**Methods:**

To address this, we propose a novel computational framework that synergistically integrates clinical prior knowledge with data-driven learning for non-invasive screening via tongue image analysis. Our framework introduces two key innovations: a TransNeXt hybrid backbone to extract comprehensive local and global tongue features, and a Channel Attention Gated Fusion module that performs asymmetric feature recalibration and prior-guided adaptive integration.

**Results:**

Evaluated on a multicenter gastrointestinal disease tongue image dataset, our framework demonstrates robust performance, with an SVM classifier achieving an accuracy of 0.874, a Macro-F1 score of 0.879, and an AUC of 0.898.

**Discussion:**

Ablation studies confirm the critical synergy between multimodal feature fusion and data augmentation. This work establishes an effective and objective computational solution for tongue diagnosis, with strong potential for clinical translation as an auxiliary screening tool.

## 1 Introduction

Gastrointestinal diseases present a formidable global health challenge, accounting for approximately 443.53 million incident cases and 2.56 million deaths annually, which represents 3.51% of total disability-adjusted life years Wang et al. (2023). In China, this burden is particularly acute; with an estimated 499.2 million cases and 1.56 million deaths Cheng et al. (2024), the country represents a substantial proportion of global GID prevalence and mortality. Conditions such as gastric polyp, chronic gastritis, and gastroesophageal reflux disease dominate this landscape due to their high incidence rates and severe complications Black et al. (2020). Although endoscopy with biopsy remains the clinical gold standard, its invasiveness, high cost, and intensive resource requirements limit its scalability for population-wide screening and routine monitoring. Consequently, there is an urgent need for non-invasive and accessible diagnostic auxiliary tool.

Tongue diagnosis, a cornerstone of Traditional Chinese Medicine (TCM) for over three millennia, provides a unique, non-invasive window into systemic health Zhang Z. et al. (2023). According to TCM theory, variations in tongue manifestations reflect underlying physiological and pathological states. Recent translational research has corroborated associations between specific tongue features and various systemic conditions, including gastric Yuan et al. (2023) and colorectal cancers Sun et al. (2025), diabetes Babu et al. (2024), and fatty liver disease Cao et al. (2023), highlighting its potential as a diagnostic proxy for GIDs. However, traditional clinical tongue diagnosis is inherently subjective Jia et al. (2024), as it relies heavily on practitioner experience, leading to significant inter-observer variability.

Recent computational advancements have sought to objectify tongue image analysis through two primary paradigms: knowledge-driven approaches based on handcrafted clinical features, and data-driven deep learning models Tiryaki et al. (2024); Kanawong et al. (2012). While the former offers high interpretability, it often lacks the representational capacity to model complex pathological variations. Conversely, deep learning models provide powerful pattern recognition with limited interpretability, hindering their clinical integration. To bridge this gap, hybrid frameworks have emerged; however, they face two fundamental limitations. First, architecturally, they typically rely on a single class of networks such as standard Convolutional Neural Networks (CNNs) or Vision Transformers (ViTs). Such single-type architectures are insufficient to simultaneously capture fine-grained local patterns and long-range global dependencies, both of which are essential for accurate tongue image analysis. Second, their integration mechanisms are frequently superficial, employing naive operations like feature concatenation. Such methods fail to reconcile the semantic and distributional disparities between handcrafted and deep-learned representations, often introducing redundancy or noise rather than achieving the synergistic fusion required for reliable diagnostic inference.

To address these challenges, we propose a novel auxiliary diagnostic framework that synergistically integrates clinical prior knowledge with advanced deep learning for robust GID screening. Unlike existing fusion methods that typically treat handcrafted and deep features as symmetric peers, our framework explicitly models the asymmetric relationship between clinical prior knowledge and datadriven representations, allowing domain knowledge to serve as a supervisory signal that guides feature integration.The key innovations of this work are as follows:

- To address the architectural limitation of single-type networks, we adopt the TransNeXt Shi (2024) hybrid backbone for deep feature extraction. Unlike models that rely solely on either convolutional or selfattention mechanisms, this hybrid design simultaneously captures fine-grained local details and long-range global dependencies, providing a more comprehensive representation tailored to tongue image analysis.
- To achieve refined integration, we introduce a Channel Attention Gated fusion module, which employs an asymmetric, modality-specific attention mechanism to separately recalibrate handcrafted and deep features, followed by a prior-guided gating strategy that adaptively modulates deep representations conditioned on the refined clinical priors. In contrast to existing fusion strategies based on simple concatenation or static weighting, our method explicitly models the semantic heterogeneity between modalities and enables knowledge-guided feature interaction.
- We construct and evaluate our framework on a multicenter gastrointestinal disease tongue image dataset, ensuring its clinical relevance and validating its effectiveness in distinguishing between multiple GIDs. Extensive experiments demonstrate that the proposed framework consistently outperforms conventional deep learning and fusion-based approaches, highlighting the effectiveness of the proposed prior-guided integration strategy.

## 2 Related work

### 2.1 Evolution of tongue image feature modeling

The tongue serves as a critical visual indicator of gastrointestinal health, positioning computer-aided tongue image analysis as a pivotal non-invasive diagnostic modality. This field has evolved along two primary technical trajectories: knowledge-driven feature engineering and data-driven representation learning. Traditional approaches rely on handcrafted features that computationally encode clinical diagnostic criteria, such as quantitative measures of color, texture, and morphology, which are subsequently fed into classifiers for disease screening Cao et al. (2023); Zhang et al. (2013). The intrinsic strength of these features lies in their interpretability, as they directly map to visual traits assessed in clinical practice. However, manual feature engineering often fails to capture complex, high-order pathological patterns, thereby limiting robustness and generalizability. The advent of deep learning has shifted the paradigm toward automated feature extraction. CNNs such as ResNet He et al. (2016) and DenseNet Huang et al. (2017) have become benchmarks for learning discriminative representations, consistently demonstrating superior performance. More recently, ViTs have been explored to model long-range spatial dependencies Jia et al. (2024); Fu et al. (2024). Despite their efficacy, these models often function as black boxes, offering limited transparency and potentially neglecting well-founded clinical priors embedded in handcrafted features. This dichotomy between interpretable but constrained handcrafted features and powerful yet opaque deep features underscore the necessity for effective fusion strategies.

### 2.2 Feature fusion in medical image analysis

Feature fusion integrates complementary information to construct robust representations, serving as a cornerstone of multi-scale medical image analysis Liu (2025). In the context of combining handcrafted and deep features, intermediate fusion is particularly salient, as it enables meaningful interactions prior to final classification. While common implementations rely on simple concatenation or coefficient weighting, advanced learnable strategies have emerged to dynamically recalibrate heterogeneous features. In tongue image analysis, preliminary studies have utilized basic fusion methods, such as merging color histograms with CNN-derived features Zubair et al. (2025). Beyond tongue analysis, a retinal disease detection study Khan et al. (2025) has also explored feature integration through a dual-module framework that captures both global context and local details, with features fused via a synergic network using concatenation and fully connected layers. More broadly, existing fusion frameworks exhibit two critical limitations: naive integration strategies fail to model complex inter-dependencies between disparate feature types; conventional backbones inadequately capture the interplay between fine-grained local details and global contextual relationships. To bridge these gaps, a framework capable of the adaptive integration of priorguided clinical features and data-driven representations is essential.

### 2.3 Tongue image–based diagnosis of gastrointestinal diseases

Tongue analysis has gained increasing traction as a cost-effective adjunct for screening GIDs. Yuan et al. Yuan et al. (2023) demonstrated the stability of tongue images in diagnosing gastric cancer, achieving Area Under the Curve (AUC) values between 0.83 and 0.88 in external validation sets. Qiao et al. (2024) integrated handcrafted features, auto-encoded representations, and spatial information to diagnose five common GIDs, achieving an accuracy of 0.849. Similarly, Gholami et al. (2021) combined deep neural networks with Support Vector Machines based on surface and color features, reaching a 0.91 accuracy for gastric cancer detection. Ma et al. (2021) further illustrated that integrating deep learning tongue features with canonical risk factors improved the screening of gastric precancerous lesions by 10.3% compared to using risk factors alone. Furthermore, Wu et al. (2020) identified specific diagnostic indices for gastroesophageal reflux disease, noting that saliva volume and fur thickness in the spleen–stomach area were significantly elevated (*p<* 0.05) in patients. Collectively, these studies substantiate that tongue manifestations harbor discriminative visual patterns essential for GID assessment.

## 3 Materials and methods

**Figure 1:**
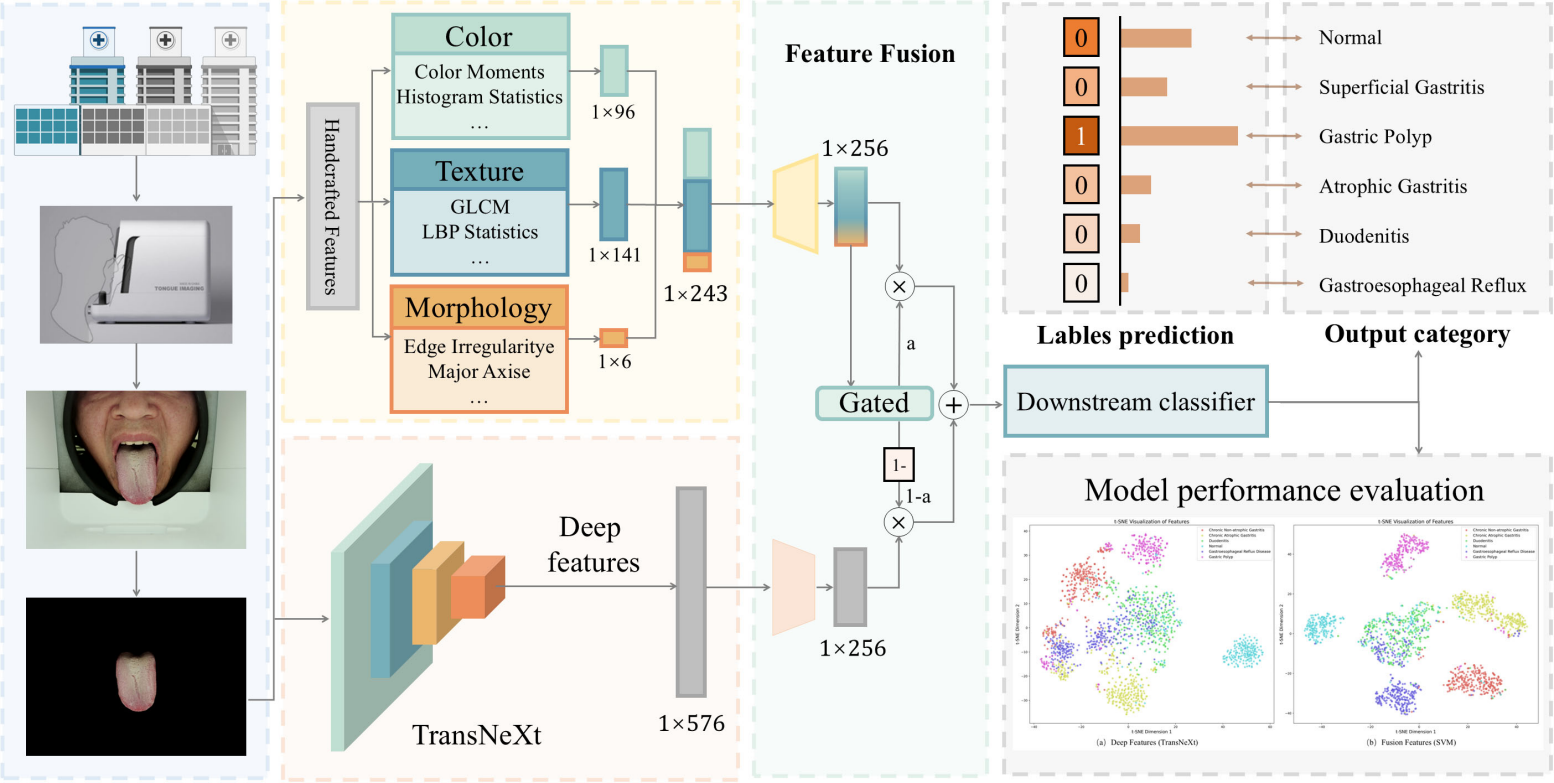
Overview of the proposed diagnostic framework. Handcrafted clinical features and deep features are extracted from tongue images, then fused via a prior-guided attention mechanism for disease classification. This design mirrors how clinicians integrate visual observations with clinical knowledge.

The overall pipeline of the proposed framework is illustrated in **Figure 1**, comprising four key stages. First, a custom Intelligent Tongue Diagnosis Instrument acquires standardized images, ensuring color fidelity. Second, multi-dimensional features are extracted from dual pathways: clinically interpretable handcrafted features and deep representations from a pre-trained TransNeXt backbone. Third, a channel attentionguided gate dynamically fuses these heterogeneous features into a unified 256-dimensional embedding. Finally, the optimal classifier is selected from six diverse models based on validation performance.

### 3.1 Study population

From September 2023 to December 2024, tongue images were prospectively collected from consecutive subjects undergoing gastroscopy at three clinical centers in Sichuan Province: the Second Hospital of Traditional Chinese Medicine of Sichuan Province, Guanghan Hospital of Traditional Chinese Medicine, and Anyue County People’s Hospital. Eligible participants met the following criteria: age between 18 and 80 years; completion of both endoscopic examination and histopathological evaluation; and for disease groups, a confirmed diagnosis of chronic non-atrophic gastritis, chronic atrophic gastritis, gastric polyps, duodenitis, or gastroesophageal reflux disease based on the International Classification of Diseases. This study was approved by the Medical Ethics Committee of the Second Hospital of Traditional Chinese Medicine of Sichuan Province (approval number: 202304(H)-003-01), with written informed consent obtained from all participants prior to enrollment.

### 3.2 Tongue image acquisition

Tongue images were captured under standardized conditions using a custom-developed Intelligent Tongue Diagnosis Instrument. The device features an enclosed dark chamber to eliminate ambient light interference, an inclined design facilitating natural tongue protrusion, and a chin rest ensuring consistent positioning. Illumination is provided by industrial-grade LED panels (BRD6030, color temperature 6000 K) calibrated for color fidelity. Images were acquired using a 20.2-megapixel camera (HELICORN 2000-IMX283, resolution 5120 × 3680 pixels). This setup ensures high reproducibility and standardization of image acquisition. Participants were instructed to sit upright, gaze straight ahead, and extend the anterior twothirds of the tongue gently with the dorsal surface fully exposed. Quality control excluded images with blur due to overexposure or defocus, incomplete tongue coverage, or severe obscuration of tongue coating due to moisture.

**Figure 2:**
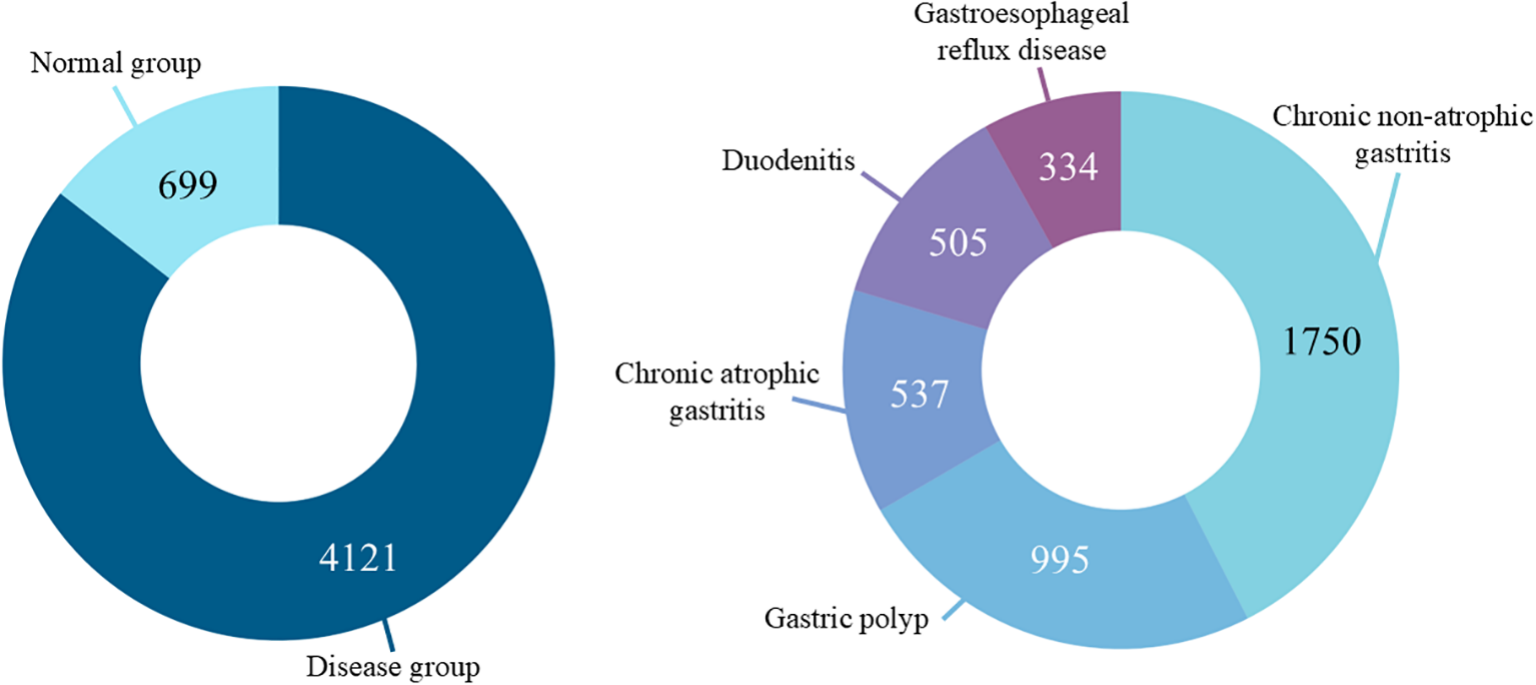
Distribution of tongue images across disease categories. The dataset exhibits class imbalance, reflecting real-world clinical populations and motivating our use of macro-averaged metrics.

After quality screening, a total of 4820 high-quality tongue images paired with corresponding gastroscopy reports were retained for analysis. As illustrated in **Figure 2**, the distribution across diagnostic categories in this dataset exhibits significant class imbalance. The entire dataset was partitioned into training, validation, and test sets at a ratio of 7:1:2, with stratification applied to preserve the original class distribution.

### 3.3 Image preprocessing pipeline

To ensure a robust input for downstream analysis, all tongue images undergo a standardized preprocessing pipeline focused on region-of-interest extraction and clinically plausible augmentation.

#### 3.3.1 Tongue region extraction and refinement

Accurate tongue segmentation is critical to eliminate interference from surrounding structures such as lips and teethWu et al. (2024). We employ the Segment Anything Model (SAM) Kirillov et al. (2023), a vision foundation model with strong zero-shot generalization capability, to achieve this. For each image, sparse foreground points are provided as positional prompts. SAM encodes these prompts into high-dimensional embeddings. These embeddings interact with visual features through a multi-layer cross-attention decoder, achieving deep alignment between prompt information and image context. This process generates segmentation masks that adhere to clinically meaningful anatomical boundaries without requiring extensive pixel-level annotations. Together, this process constitutes an efficient weakly supervised paradigm. Subsequently, a boundary refinement step is applied. Given the binary mask *M*, we compute the minimum bounding rectangle *R* enclosing all coordinates (*x,y*) where *M*(*x,y*) = 1, and crop the original image accordingly. This removes uninformative black borders while preserving all tongue-related information, yielding a clean region of interest for downstream analysis.

#### 3.3.2 Clinically plausible data augmentation

To address the issues of class imbalance and limited training sample size, this study drew upon the method described in Yao et al. (2024) and designed a data augmentation strategy based on clinical practice. This strategy incorporates reasonable geometric and photometric transformations, which reflect common acquisition inconsistencies: random small-angle rotations (± 15°), horizontal flipping, mild Gaussian blurring, and small-scale random translations. Each training image is randomly applied with combinations of these transformations to generate diverse and anatomically realistic variants. This process enhances the model’s robustness while strictly preserving the critical diagnostic features. All augmentation operations are limited to the training set to prevent data leakage.

### 3.4 Feature extraction

#### 3.4.1 Handcrafted feature extraction

In TCM, tongue color, morphology, moisture, and texture provide critical diagnostic insights. Computational tongue diagnosis quantifies these visual cues to enable objective assessment. For gastrointestinal diseases, specific signs are clinically indicative: yellowish or greasy coating suggests damp-heat; dark purple tongue implies blood stasis; scalloped edges reflect qi deficiency; prominent fissures indicate chronic pathology. Guided by these principles, we operationalize qualitative observations into three interpretable feature sets: color, texture, and morphology, as summarized in **Table 1**.

**Table 1:** Summary of handcrafted tongue image features.

Feature type	Feature name	Feature description
Color	Color Moments Histogram Statistics	Mean, variance, skewness in RGB, HSV, CIELAB, YCbCr Mean, variance, energy, contrast, entropy from 64-bin histograms
Texture	GLCM	Contrast, Dissimilarity, Homogeneity, Energy, Correlation, ASM, based on 4 directions and 3 distances
	LBP	Uniform LBP (*P* = 8) at radii 1–3; 18-bin histogram per scale
	LBP Statistics	Histogram-derived mean, variance, energy, contrast, entropy
Morphology	Edge Irregularity Major Axis Minor Axis Aspect Ratio	Perimeter–area complexity descriptor Major axis length obtained via ellipse fitting Minor axis length obtained via ellipse fitting Ratio of major axis length to minor axis length
	Circularity	Calculated as 4*π* · area*/*perimeter^2^
	Area Ratio	Tongue to total image area proportion

Color Feature Extraction: To comprehensively characterize color distribution, we extract low-level statistical features across four color spaces Zhang N. et al. (2023): RGB, HSV, CIELAB, and YCbCr. For each channel, we compute mean, variance, and skewness (36 dimensions). Additionally, 64-bin histograms are constructed, from which energy, contrast, entropy, mean, and variance are derived (60 dimensions). The concatenated 96-dimensional vector provides complementary representations for classification.

Texture Feature Extraction: Texture is quantified using Gray-Level Co-occurrence Matrix (GLCM) Sebastian V et al. (2012) and Local Binary Patterns (LBP). GLCM is computed at four orientations (0°, 45°, 90°, 135°) and three distances (1, 3, 5 pixels) and three distances (1, 3, 5 pixels). For each of the 12 configurations, we extract six texture descriptors: contrast, dissimilarity, homogeneity, energy, correlation, and angular second moment, yielding 72 dimensions. For LBP, we generate uniform patterns using 8 points at radii of 1, 2, and 3 pixels. At each scale, we compute an 18-bin histogram and derive mean, variance, energy, contrast, and entropy, resulting in 69-dimensional multi-scale LBP. The fused 141-dimensional texture vector captures both spatial correlations and structural variations.

Morphological Feature Extraction: Geometric properties are quantified through six descriptors derived from the segmented region. Edge irregularity is defined as *P*^2^*/A*, where *P* and *A* denote the perimeter and area, respectively. Aspect ratio reflects tongue elongation via the ratio of major to minor axis lengths from ellipse fitting. Circularity is given by 4*πA/P*^2^, measuring shape approximation to a perfect circle. Area ratio characterizes relative tongue occupancy within the field of view. Additionally, absolute length and width are included as complementary dimensional measures. These six descriptors enhance sensitivity to clinically meaningful shape variations.

Collectively, the proposed handcrafted feature set **f***_h_*comprises 243 dimensions (96 color + 141 texture + 6 morphology), translating TCM diagnostic criteria into a reproducible computational framework.

#### 3.4.2 Deep feature extraction

**Figure 3:**
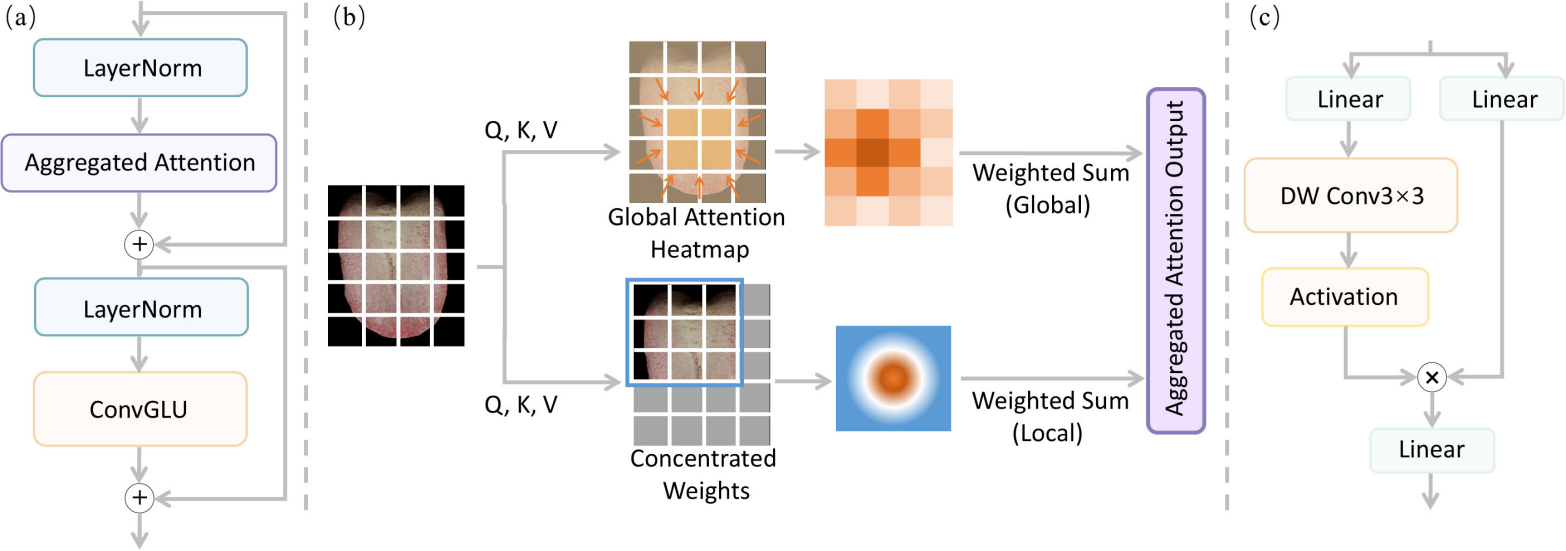
Architecture of a TransNeXt block. **(a)** Overall structure of the block. **(b)** Aggregated Attention combines local and global visual information. **(c)** The Convolutional Gated Linear Unit performs channel-wise recalibration to highlight clinically meaningful texture and color patterns.

To extract high-level discriminative representations from tongue images, we employ TransNeXt-Tiny as the deep feature encoder. As shown in **Figure 3a**), TransNeXt is a hierarchical Vision Transformer incorporating Aggregated Attention (AA) and Convolutional Gated Linear Units (ConvGLU). Given the heterogeneous textures and spatially distributed diagnostic cues in tongue images, this design is especially well-suited for capturing both local details and global context.

The input tongue image is transformed by an overlapping patch embedding stem into an initial feature map $X\in {\mathbb{R}}^{C\times H^{\prime }\times W^{\prime }}$. Within each transformer stage, the feature map undergoes two sequential operations: ConvGLU-based channel recalibration followed by Aggregated Attention-based token mixing. To capture both local and global visual patterns, the Aggregated Attention computes two sets of similarity scores (see **Figure 3b**): S_local_ for the local sliding window and S_global_ for the globally pooled context. At each spatial location (*i,j*), these scores are computed as (Equations 1, 2):

(1) $$S_{\text{local}}=\left({\hat{Q}}_{i,j}+Q_{E}\right){\hat{K}}_{\rho }^{⊤},$$

(2) $$S_{\text{global}}=\left({\hat{Q}}_{i,j}+Q_{E}\right){\hat{K}}_{\sigma }^{⊤},$$

where $\hat{Q}$ and $\hat{K}$ represent $\ell_{2}$-normalized query and key projections, and Q*_E_* denotes a learnable query 215 embedding shared across spatial positions. The aggregated attention weights are obtained via a unified 216 softmax (Equation 3):

(3) $$A_{i,j}=\text{softmax}\;(\tau \text{log }N_{i,j}\cdot \left[S_{\text{local}}∥S_{\text{global}}\right]+B_{i,j}),$$

where *N_i,j_* is the effective number of valid tokens. The learnable scaling factor *τ* controls the sharpness of attention, and the positional bias B*_i,j_* ensures spatial consistency. This equation integrates local and global similarity scores into a unified attention distribution, enabling the model to focus on clinically relevant regions at multiple spatial scales. The attention weights are then split as $A_{i,j}=\left[A_{i,j}^{\rho },A_{i,j}^{\sigma }\right]$, corresponding to local and global token sets, respectively. The final aggregated attention output at position (*i*, *j*) is computed as (Equation 4):

(4) $$\text{AA}\left(X_{i,j}\right)=(A_{\text{local}}+{\hat{Q}}_{i,j}T)\;V_{\text{local}}+A_{\text{global}}V_{\text{global}},$$

where $T\in {\mathbb{R}}^{d\times k^{2}}$ denotes a set of learnable positional tokens. This component ${\hat{Q}}_{i,j}T$ enables query dependent positional modulation, allowing the model to adapt its focus based on the specific region being analyzed. In essence, the AA mechanism acts like a clinician who shifts attention between a suspicious spot and the overall tongue appearance, capturing both fine-grained details and broader patterns in a unified manner. Following token mixing, channel-wise feature recalibration is performed using ConvGLU as (Equation 5):

(5) $$\text{ConvGLU}\left(X\right)=W_{1}\;(X⊙\text{GELU}\;\left({\text{DWConv}}_{3\times 3}\;(W_{2}X)\right)),$$

where W_1_, W_2_ are linear projections, DWConv_3×3_(·) denotes depthwise convolution, and ⊙ denotes element-wise multiplication. The ConvGLU module performs channel-wise feature recalibration by using depthwise convolution to capture local spatial patterns per channel, then selectively emphasizing informative channels via element-wise multiplication. This enhances the model’s ability to highlight clinically meaningful texture and color patterns in the tongue image.

After four hierarchical stages, the output feature map from the final stage $X^{\left(S\right)}\in {\mathbb{R}}^{C_{S}\times H_{S}\times W_{S}}$ is spatially aggregated via global average pooling to yield a compact deep representation (Equation 6):

(6) $$f_{d}=\frac{1}{H_{S}W_{S}}\sum_{i=1}^{H_{S}}\sum_{j=1}^{W_{S}}X_{i,j}^{\left(S\right)}.$$

This operation compresses spatial dimensions while preserving salient channel-wise information, yielding a fixed-length vector that captures overall tongue image characteristics. For TransNeXt-Tiny pretrained on ImageNet-1K with an input resolution of 224 × 224, the final-stage feature map has *C_S_*= 576 channels and spatial dimensions *H_S_*= *W_S_*= 7. This yields a 576-dimensional feature vector **f***_d_*, serving as the deep tongue representation for subsequent diagnostic modeling or feature fusion analysis.

### 3.5 Feature Fusion

To enable adaptive integration while preserving modality-specific semantics, we propose Channel

**Figure 4:**
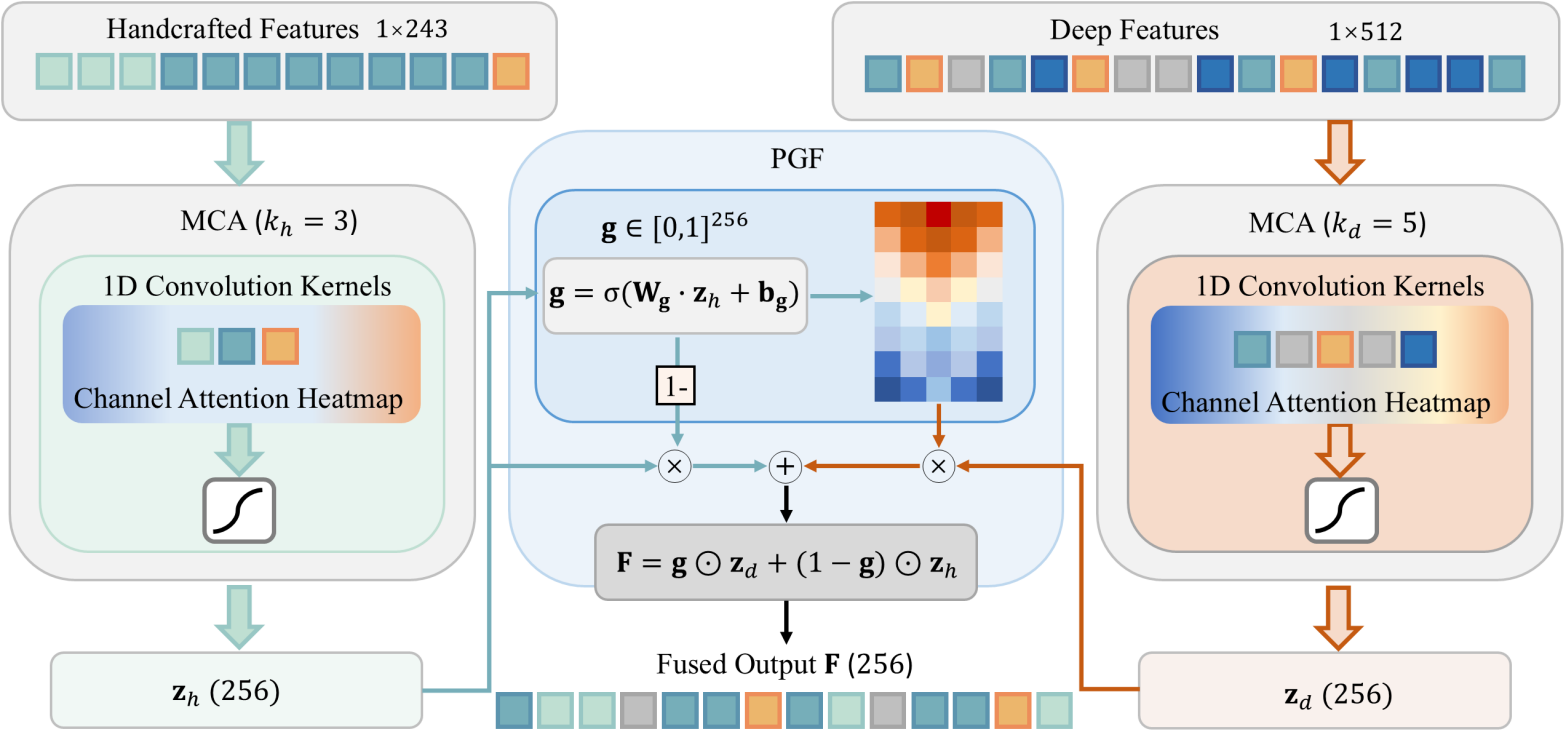
Schematic diagram of the channel attention gate fusion module. Handcrafted features are used as prior knowledge to modulate deep features via an asymmetric gating mechanism, ensuring that clinically established indicators guide the final diagnosis.

Attention Gated fusion (CA-Gate) module. As shown in **Figure 4**, this module incorporates a two-stage fusion mechanism: Modality-specific Channel Attention (MCA) recalibration via efficient 1D channel attention, and Prior-guided Gated Fusion (PGF). Let $f_{h}\in {\mathbb{R}}^{d_{h}}$ and $f_{d}\in {\mathbb{R}}^{d_{d}}$ denote the handcrafted and deep feature vectors extracted from a tongue image, where *d_h_*= 243 and *d_d_*= 576.

#### 3.5.1 Modality-specific channel attention recalibration

To account for the heterogeneous semantic granularity between handcrafted and deep representations, we employ modality-specific channel attention with distinct kernel sizes. Each feature stream undergoes independent channel attention to suppress irrelevant dimensions and amplify diagnostically salient cues.

The refined representations ${\tilde{f}}_{h}$ and ${\tilde{f}}_{d}$ are computed as (Equations 7, 8):

(7) $${\tilde{f}}_{h}=f_{h}⊙\sigma \left(\text{Conv}1{\text{D}}_{k_{h}}\left(f_{h}\right)\right),$$

(8) $${\tilde{f}}_{d}=f_{d}⊙\sigma \left(\text{Conv}1{\text{D}}_{k_{d}}\left(f_{d}\right)\right),$$

where *σ*(·) is the sigmoid activation function that produces channel-wise scaling weights, and Conv1D*_k_*(·) represents a 1D convolution along the channel dimension with kernel size *k*.

Given that handcrafted features consist of compact, interpretable attribute groups, a smaller kernel (*k_h_*= 3) suffices to capture local intra-group dependencies. In contrast, deep features from TransNeXt exhibit richer cross-channel semantics spanning multiple diagnostic concepts, warranting a larger receptive field along the channel dimension; thus, *k_d_*= 5 is adopted. This asymmetric design enables context-aware recalibration aligned with the statistical properties of each modality. The refined features ${\tilde{f}}_{h}$ and ${\tilde{f}}_{d}$ are then projected into a shared latent space of dimension *d_z_*= 256 as (Equations 9, 10):

(9) $$z_{h}=W_{p}^{\left(h\right)}\cdot {\tilde{f}}_{h}+b_{p}^{\left(h\right)},\;W_{p}^{\left(h\right)}\in {\mathbb{R}}^{d_{z}\times d_{h}},$$

(10) $$z_{d}=W_{p}^{\left(d\right)}\cdot {\tilde{f}}_{d}+b_{p}^{\left(d\right)},\;W_{p}^{\left(d\right)}\in {\mathbb{R}}^{d_{z}\times d_{d}},$$

where $W_{p}^{\left(h\right)},W_{p}^{\left(d\right)}$ and biases $b_{p}^{\left(h\right)},b_{p}^{\left(d\right)}$ constitute linear projection layers. This compact dimensionality is chosen to balance representational capacity and generalization: while sufficiently expressive to preserve discriminative cues from both modalities, it avoids excessive parameterization that could lead to overfitting in our clinical dataset of limited scale. The resulting aligned embeddings $z_{h},z_{d}\in {\mathbb{R}}^{d_{z}}$ serve as inputs to the subsequent gated fusion stage.

#### 3.5.2 Prior-guided Gated Fusion

To realize knowledge-informed integration, the refined handcrafted embedding **z***_h_* is leveraged as a diagnostic prior to modulate the data-driven deep representation **z***_d_*. Specifically, a learnable gating mechanism generates an adaptiveweight vector $g\in {\left[0,1\right]}^{d_{z}}$ conditioned solely on **z***_h_* as (Equation 11):

(11) $$g=\sigma \left(W_{g}\cdot z_{h}+b_{g}\right),$$

where *σ*(·) ensures soft gating. This equation generates a gating vector based on handcrafted features, which serve as clinical priors to modulate the contribution of deep features. The final fused embedding $F\in {\mathbb{R}}^{d_{z}}$ is computed as a convex combination of the two refined streams (Equation 12):

(12) $$F=g⊙z_{d}+\left(1-g\right)⊙z_{h}.$$

This equation adaptively combines deep features and handcrafted clinical features, where the gating vector g determines the contribution of each modality. In this way, clinically interpretable features guide the integration process, ensuring that domain knowledge informs the final representation. For instance, when morphological indicators suggest a specific syndrome, the gate amplifies relevant deep semantic channels while suppressing potentially misleading data-driven responses. Notably, the fusion process is asymmetric: the handcrafted features guide the deep features, but not vice versa. This design preserves the supervisory role of domain knowledge, treating it as a reference standard rather than a peer modality, a valuable choice in medical applications where clinical expertise should anchor the learning process.

### 3.6 Downstream classifier

To validate the generalizability and discriminative power of the fused representation, we evaluate six widely adopted machine learning classifiers spanning diverse learning paradigms: Support Vector Machine (SVM) Cortes and Vapnik (1995), Logistic Regression (LR), Random Forest (RF)Breiman (2001), K-Nearest Neighbors (KNN), Multi-Layer Perceptron (MLP) and XGBoost Chen and Guestrin (2016). The hyperparameters of all models are selected by grid search on a held-out validation set to maximize macro-averaged F1 score, with detailed search spaces provided in Supplementary Material **Supplementary Table 1**.

Notably, the same unified embedding is fed into all classifiers, demonstrating that CA-Gate produces a modality-agnostic representation compatible with both classical statistical models and modern ensemble or neural approaches. The classifier achieving the best validation performance is selected as the final diagnostic model.

### 3.7 Implementation details

All experiments were conducted on an NVIDIA GeForce RTX 4090 GPU using Python 3.8.19 and PyTorch 2.3.1. Deep feature extraction models were initialized with ImageNet pre-trained weights and optimized using stochastic gradient descent with an initial learning rate of 1×10^−3^, momentum of 0.9, and weight decay of 5×10^−4^. The learning rate was decayed using cosine annealing to 1×10^−5^ over 300 epochs with a batch size of 32. Dropout with *p* = 0.1 was applied during feature extraction and fusion stages; early stopping was triggered when validation macro-averaged F1 score (Macro-F1) showed no improvement for 20 consecutive epochs. For traditional classifiers (scikit-learn), optimal hyperparameters were determined via grid search on the training set. The held-out test set was used strictly for final performance reporting.

Given class imbalance in our dataset, we adopt Macro-F1 as the primary metric, computed as the unweighted average of per-class F1 scores. Secondary metrics include accuracy, precision, recall, specificity, AUC using one-vs-rest strategy, and Cohen’s Kappa coefficient. All metrics are computed on the test set using the selected checkpoint.

## 4 Experiments and results

### 4.1 Comparison of deep feature extraction models

**Figure 5:**
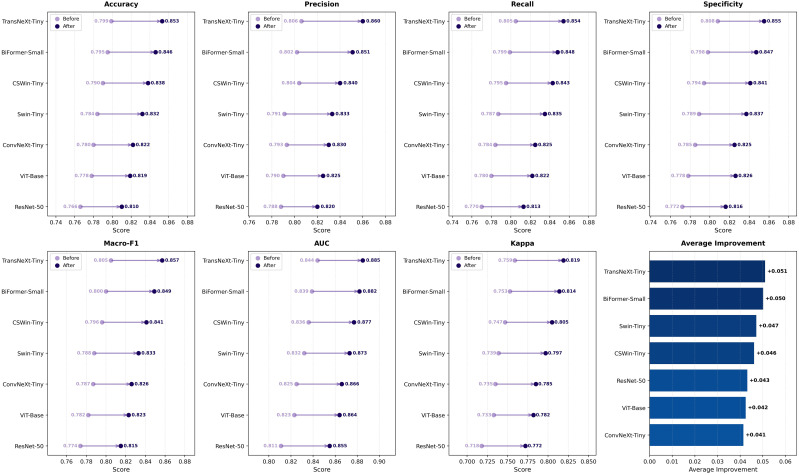
Performance comparison of seven deep learning models before and after data augmentation. Lollipop plots illustrate the improvement across seven evaluation metrics, with the bar chart showing average improvement across all metrics. TransNeXt achieves the highest performance, and augmentation strategy consistently improves results, highlighting the value of training diversity for real-world generalization.

To identify an effective backbone for tongue image representation, seven state-of-the-art deep architectures were evaluated under identical training protocols and data splits. As shown in **Figure 5**, TransNeXt-Tiny achieves the highest performance across all evaluation metrics, including an accuracy of 0.853, a Macro-F1 score of 0.857, an AUC of 0.885, and a Cohen’s Kappa of 0.819. Compared with other competitive architectures, TransNeXt-Tiny demonstrates consistent improvements in robust metrics. In particular, it outperforms BiFormer-Small Zhu et al. (2023) by 0.008 in Macro-F1 and 0.003 in AUC, and shows clear margins over Swin-Tiny (+0.024) Liu et al. (2021), ConvNeXt-Tiny (+0.021) Liu et al. (2022), and CSWin-Tiny (+0.016) Dong et al. (2022) in Macro-F1. The high Kappa score further indicates strong agreement between model predictions and clinical annotations, reflecting reliable diagnostic consistency.

To assess whether these improvements are statistically significant and robust to training variability, we conducted five independent training runs for each backbone using different random seeds while keeping all hyperparameters and data splits identical. This allows us to quantify the uncertainty arising from stochastic factors such as weight initialization and mini-batch shuffling. For each model, we computed the mean Macro-F1 and its 95% confidence interval (CI) based on the t-distribution, which is appropriate for small sample sizes. As summarized in **Table 2**, TransNeXt-Tiny achieved the highest mean Macro-F1 (0.858, 95% CI: 0.853–0.862), with the narrowest CI among all backbones, indicating both superior performance and high stability. To directly compare model pairs, we performed paired t-tests on the five run-wise Macro-F1 scores, which eliminate inter-run variability and isolate the relative performance difference.

**Table 2:** Comparison of deep feature extraction models, reporting Macro-F1 scores across five independent training runs along with statistical summaries.

Model	Run 1	Run 2	Run 3	Run 4	Run 5	Mean	Std	95% CI
ResNet-50	0.819	0.815	0.802	0.821	0.817	0.815	0.007	(0.805, 0.824)
ConvNeXt-Tiny	0.824	0.821	0.832	0.825	0.829	0.826	0.004	(0.821, 0.832)
ViT-Base	0.829	0.826	0.815	0.821	0.825	0.823	0.005	(0.816, 0.830)
Swin-Tiny	0.833	0.839	0.824	0.835	0.831	0.832	0.006	(0.826, 0.839)
CSWin-Tiny	0.839	0.844	0.837	0.841	0.846	0.841	0.004	(0.837, 0.846)
BiFormer-Small	0.846	0.848	0.856	0.843	0.850	0.849	0.005	(0.843, 0.855)
TransNeXt-Tiny	0.853	0.857	0.856	0.863	0.859	0.858	0.004	(0.853, 0.862)

Values for individual runs are reported as raw scores; statistical summaries include mean, standard deviation (Std), and 95% confidence interval (CI) computed via the t-distribution.

The results showed that TransNeXt-Tiny significantly outperformed ResNet-50 (*p* = 1.81 × 10^−4^), SwinTiny (*p* = 6.86 × 10^−4^) and Biformer-Small (*p* = 4.86 × 10^−2^). These findings confirm that the observed performance advantages are not attributable to random fluctuations and remain consistent across different training conditions.

The overall performance advantage of TransNeXt-Tiny suggests that its aggregated attention mechanism and convolution-enhanced channel modeling are effective in capturing discriminative tongue patterns across multiple diagnostic categories. Based on these results, TransNeXt-Tiny is selected as the deep feature extractor in the proposed hybrid framework.

### 4.2 Comparative evaluation of downstream classifier

**Figure 6:**
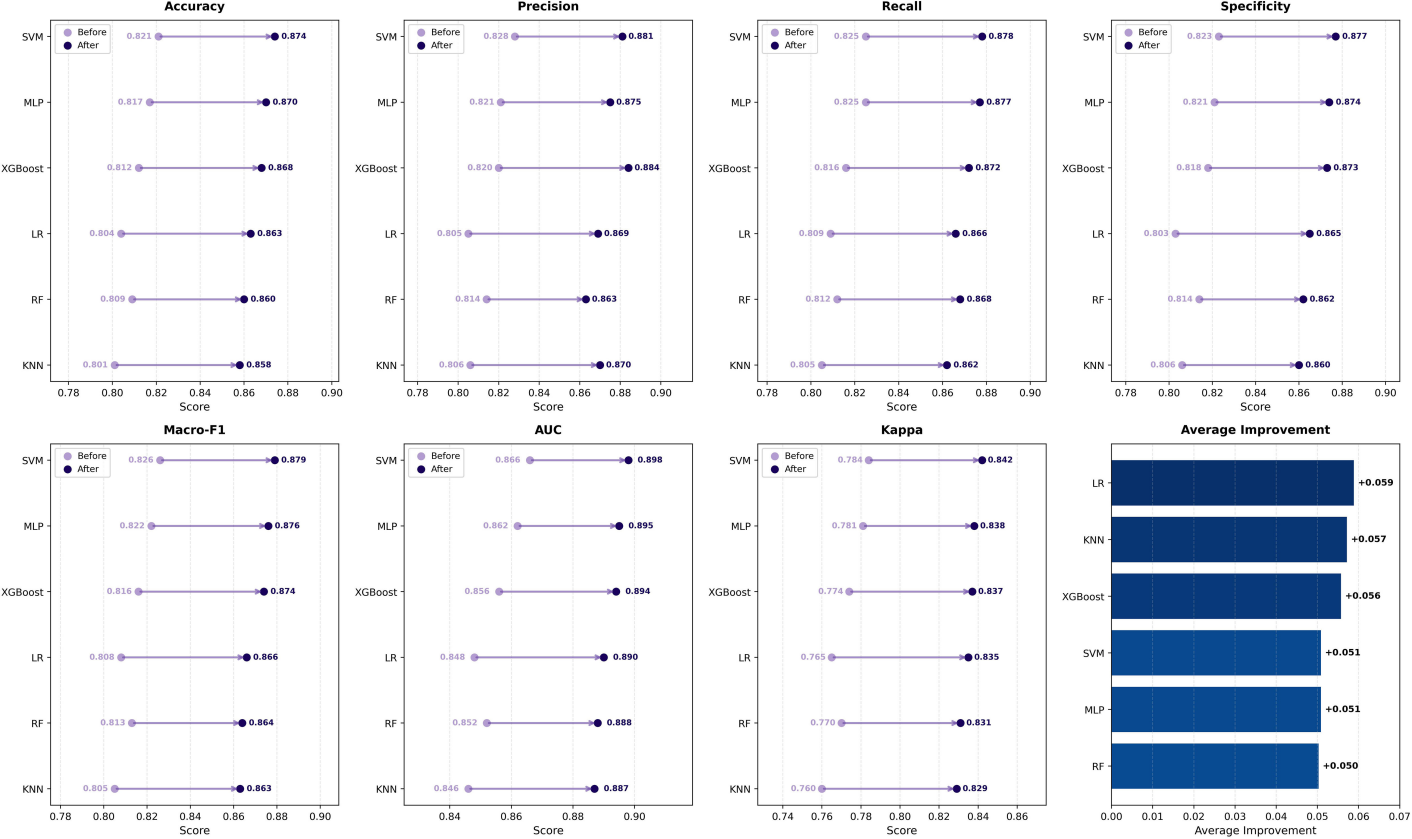
Performance comparison of six downstream classifiers before and after data augmentation, based on seven evaluation metrics.

The performance of various downstream classifiers, evaluated using the fused feature representations, is quantitatively summarized in **Figure 6**. SVM achieves the best overall performance, attaining the highest accuracy of 0.874, Macro-F1 of 0.879, AUC of 0.898, and Cohen’s kappa of 0.842, with top rankings in six of seven metrics. MLP demonstrates competitive performance with comparable scores in accuracy (0.870) and Macro-F1 (0.876), though marginally inferior to SVM across most criteria. XGBoost ranks third, exhibiting particularly strong precision of 0.884 but relatively modest recall compared to the top performers. Notably, LR outperforms RF suggesting the fused feature space possesses enhanced linear separability that favors simpler decision boundaries over ensemble strategies. KNN exhibits the lowest performance across all metrics, likely due to its sensitivity to high-dimensional feature spaces. Given SVM’s consistent superiority and robust generalization, it is selected as the downstream classifier for all subsequent experiments.

### 4.3 Impact of feature composition on diagnostic performance

We integrate multiple handcrafted feature combinations with deep features extracted using TransNeXt and evaluate their performance in the classification of GID tongue images. As shown in **Table 3**, among single-feature configurations, texture features yielded the strongest results, with a Macro-F1 of 0.829 and an AUC of 0.868, while morphology features alone exhibited comparatively lower performance. In two-feature fusion, the combination of color and texture achieved the most balanced outcome, reaching a Macro-F1 of 0.831, which reflects effective complementarity between these feature types. Although morphology features were less discriminative in isolation, they contributed supplementary information when fused with either color or texture. The integration of all three handcrafted features led to further improvement, attaining a Macro-F1 of 0.834. Notably, incorporating deep features alongside these handcrafted ones resulted in a substantial performance gain across all evaluated metrics. This confirms that deep features effectively enhance handcrafted representations and that multimodal fusion plays a critical role in advancing diagnostic accuracy.

**Table 3:** Comparison of classification performance based on different feature combinations.

Features	Accuracy	Precision	Recall	Specificity	Macro-F1	AUC	Kappa
Color	0.813	0.814	0.819	0.817	0.815	0.859	0.775
Texture	0.825	0.838	0.828	0.832	0.829	0.868	0.789
Morphology	0.770	0.782	0.775	0.783	0.777	0.812	0.723
Color + Texture	0.829	0.834	0.830	0.829	0.831	0.870	0.795
Color + Morphology	0.818	0.819	0.825	0.820	0.821	0.862	0.783
Texture + Morphology	0.821	0.832	0.825	0.828	0.827	0.866	0.785
Color + Texture + Morphology	0.830	0.831	0.838	0.833	0.834	0.871	0.795
Color + Texture + Morphology + Deep	0.874	0.881	0.878	0.877	0.879	0.898	0.842

### 4.4 Analysis of feature space separability

**Figure 7:**
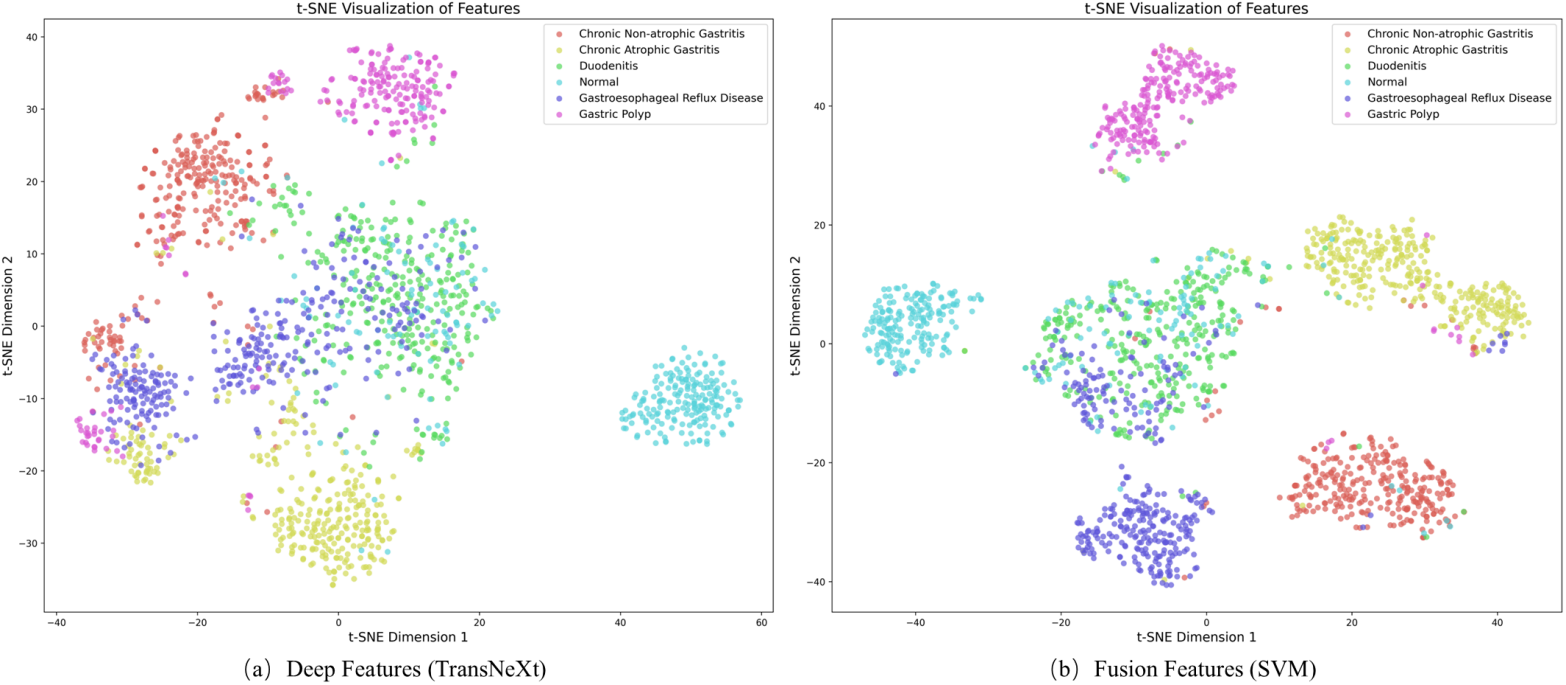
Visualization comparison of feature representations via t-SNE projection. Each point represents a tongue image. **(a)** Deep features alone show substantial overlap between disease groups. **(b)** After fusion with handcrafted features, clusters become more distinct, indicating better differentiation of similar gastrointestinal conditions.

To visually assess feature discriminability, we project the high-dimensional representations into 2D space using t-SNE. As shown in **Figure 7**, the TransNeXt-only features (a) exhibit partial separation among categories such as Chronic Non-atrophic Gastritis and Chronic Atrophic Gastritis, yet noticeable overlap persists between Gastroesophageal Reflux Disease, and between Duodenitis and Gastric Polyp. In contrast, the fused features combining TransNeXt with handcrafted color, texture, and morphology descriptors (b) form more compact and distinct clusters, with clearer separation, especially for the aforementioned overlapping classes. This structural improvement visually corroborates that feature fusion enhances class separability, which underscores the clinical relevance of the integrated representation.

### 4.5 Ablation study

#### 4.5.1 Effectiveness of data augmentation strategy

Data augmentation is adopted based on the motivation that deep feature extraction networks are highly sensitive to data diversity and tend to overfit when trained on limited tongue image samples. To evaluate its effectiveness, quantitative comparisons are conducted under two settings: end-to-end deep feature extraction models (**Figure 5**), and downstream machine learning classifiers trained on fused deep and handcrafted features (**Figure 6**).

For deep feature extraction networks, data augmentation consistently improves performance across all models and evaluation metrics. On average, Accuracy, Macro-F1, AUC, and Cohen’s Kappa are improved by 5.9%, 5.7%, 5.1%, and 7.4%, respectively, indicating enhanced representation robustness and inter-class agreement. Transformer-based architectures exhibit larger performance gains compared to CNN-based models. This disparity stems from the self-attention mechanism’s superior capability to model global contextual relationships and data-dependent interactions. Notably, TransNeXt-Tiny and BiFormer-Small achieve the highest average improvements, underscoring the effectiveness of advanced attention mechanisms in leveraging augmented data.

After fusing deep and handcrafted features, data augmentation continues to yield consistent performance gains across all downstream classifiers. As shown in **Figure 6**, the average improvements reach 6.8% in Accuracy, 6.7% in Macro-F1, 4.3% in AUC, and 8.2% in Cohen’s Kappa. Instance-based and linear classifiers (KNN and LR) exhibit the largest performance gains, whereas margin-based classifiers such as SVM and MLP show relatively moderate improvements. This pattern suggests that the fused feature space possesses enhanced linear separability and local consistency, thereby benefiting simpler decision boundaries more substantially than complex nonlinear models. The consistently lower AUC improvements across all classifiers (3.7%–5.0%) indicate that data augmentation primarily enhances classification confidence rather than ranking capability in the fused feature space. These results demonstrate that the benefits of data augmentation are effectively preserved after feature fusion, with the magnitude of improvement being classifier-dependent and inversely correlated with model complexity.

Overall, these results confirm that data augmentation enhances deep feature learning and propagates its benefits to subsequent feature fusion and classification stages. This combined effect improves the robustness of the proposed tongue image–based diagnostic framework. 4.5.2 Effectiveness of Channel Attention Gated Fusion Module

##### 4.5.2.1 Comparison with other feature fusion methods

To validate the design choices of the proposed CA-Gate module, we conducted comprehensive ablation experiments comparing alternative fusion strategies, with quantitative results summarized in **Table 4**. Direct concatenation yields the lowest performance across all metrics, indicating that naive feature stacking fails to exploit the complementary nature of heterogeneous modalities and may introduce redundancy. Learnable weighted sum achieves moderate improvements with Macro-F1 of 0.857, yet its static global weighting lacks the dynamic channel-wise adaptability required for selective cross-modal integration. Ablation of individual components reveals their distinct contributions. Removing the Modalityspecific Channel Attention degrades Macro-F1 by 0.017, underscoring the necessity of context-aware recalibration aligned with modality-specific semantic granularity. Substituting the Prior-guided Gated Fusion with simple concatenation reduces Macro-F1 by 0.006, while replacing it with learnable weighted parameters incurs a 0.002 drop, approaching but not surpassing the full CA-Gate design. The progressive performance degradation across ablated variants demonstrates that both MCA recalibration and PGF contribute indispensably. MCA ensures modality-appropriate feature refinement, while PGF enables knowledge-informed adaptive integration. The asymmetric gating mechanism, leveraging handcrafted priors to modulate deep features, proves more effective than symmetric fusion strategies, validating our design principle of preserving domain knowledge as a supervisory signal rather than treating both modalities as peers.

**Table 4:** Comparison of classification performance based on different feature fusion methods.

Method	Accuracy	Precision	Recall	Specificity	Macro-F1	AUC	Kappa
Concat	0.847	0.855	0.850	0.852	0.851	0.883	0.816
Learnable Weighted Sum	0.855	0.856	0.860	0.858	0.857	0.886	0.824
w/o MCA	0.859	0.866	0.862	0.861	0.862	0.887	0.829
Replace PGF with Concat	0.865	0.871	0.877	0.875	0.873	0.891	0.832
Replace PGF with Weighted Parameters	0.871	0.875	0.882	0.876	0.877	0.896	0.838
CA-Gate (Ours)	0.874	0.881	0.878	0.877	0.879	0.898	0.842

##### 4.5.2.2 Ablation study on convolution kernel sizes

To validate the design rationale of employing asymmetric kernel sizes in the MCA module, we conduct an ablation study by varying the combinations of (*k_h_*, *k_d_*). As shown in **Table 5**, the proposed asymmetric configuration (*k_h_*= 3*,k_d_*= 5) achieves superior performance across all evaluation metrics. In comparison, symmetric kernel settings and reversed asymmetric combination yield inferior results. These findings support our hypothesis that a smaller receptive field is preferable for compact handcrafted features, whereas a larger receptive field is necessary to capture rich cross-channel semantics in deep features. The observed performance drops in other combination methods underscores the importance of tailoring kernel sizes to the intrinsic structure of each modality.

**Table 5:** Impact of convolution kernel sizes in MCA on classification performance.

(k*_h_*,k*_d_*)	Accuracy	Precision	Recall	Specificity	Macro-F1	AUC	Kappa
(3,3)	0.866	0.870	0.874	0.872	0.872	0.891	0.832
(5,3)	0.865	0.875	0.872	0.876	0.873	0.891	0.831
(5,5)	0.869	0.876	0.874	0.872	0.874	0.893	0.835
(3,5) (Ours)	0.874	0.881	0.878	0.877	0.879	0.898	0.842

##### 4.5.2.3 Ablation study on projection dimension

**Figure 8:**
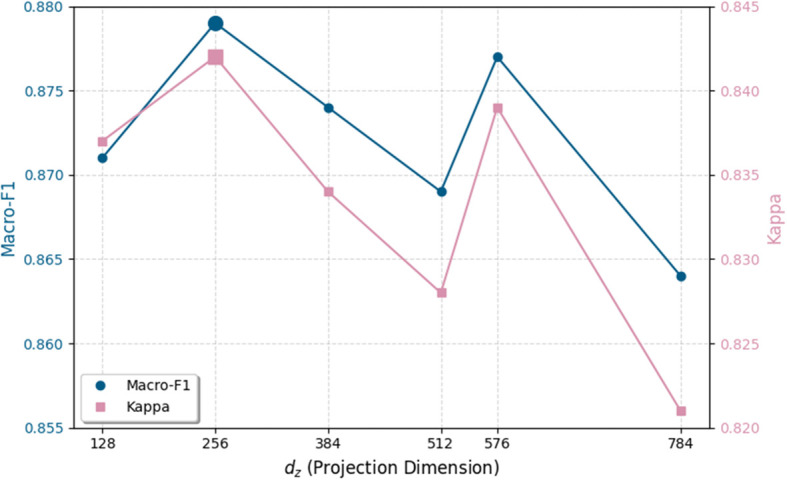
Impact of projection dimensions in MCA on classification performance. Performance peaks at *d_z_*= 256, balancing discriminative power with generalization for reliable diagnosis across patient groups.

To further investigate the impact of projection dimension on classification performance, an ablation study was conducted across six settings. As illustrated in **Figure 8**, both metrics exhibit a dependence on the chosen projection dimension. When *d_z_*increases from 128 to 256, the classification performance consistently improves and reaches its peak. Further increasing the dimensionality beyond this point results in a continuous performance degradation, indicating that inappropriate feature dimensionality may impair class separability. Although a slight performance recovery is observed at 576, both metrics decline again at 784, suggesting increased redundancy and potential overfitting in high-dimensional projections. Experimental results demonstrate that an appropriate projection dimension is critical for retaining discriminative information in the fused feature space. Based on this trade-off, 256 was selected as the optimal projection dimension.

### 4.6 Cross-center generalization validation

To evaluate whether the proposed framework generalizes across different clinical centers, we performed leave-one-center-out (LOCO) cross-validation. The dataset comprises three clinical centers: Center 1 (the Second Hospital of Traditional Chinese Medicine of Sichuan Province), Center 2 (Guanghan Hospital of Traditional Chinese Medicine), and Center 3 (Anyue County People’s Hospital). In each LOCO experiment, one center was held out as the test set, while the remaining two centers were combined and then stratified split into training and validation sets at an 8:2 ratio, preserving the original class distribution in both subsets. Data augmentation was applied only to the training set; the validation and test sets remained unaltered to preserve their original distributions.

We compared two variants: (1) TransNeXt-only, which uses TransNeXt as an end-to-end classifier, and (2) the full framework, which integrates handcrafted features via the CA-Gate module and feeds the fused representation into an SVM classifier. Both variants were retrained for each LOCO split. **Table 6** summarizes the results.The full framework consistently outperformed the TransNeXt-only variant across all three held-out centers, achieving a mean Macro-F1 of 0.877 compared to 0.856. These results suggest that incorporating handcrafted clinical features enhances cross-center generalization and that the model captures disease-relevant patterns rather than center-specific artifacts.

**Table 6:** Cross-center generalization performance comparison between TransNeXt-only and the full framework.

Method	Test Center	Accuracy	Precision	Recall	Specificity	Macro-F1	AUC	Kappa
TransNeXt-only	Center 1 Center 2	0.861 0.849	0.869 0.858	0.865 0.853	0.863 0.852	0.866 0.854	0.889 0.883	0.832 0.819
	Center 3	0.842	0.846	0.854	0.849	0.848	0.879	0.809
Full Framework	Center 1 Center 2	0.884 0.871	0.890 0.874	0.885 0.878	0.886 0.875	0.888 0.872	0.906 0.897	0.851 0.841
	Center 3	0.864	0.870	0.875	0.874	0.871	0.889	0.830

### 4.7 Visual analysis

To enhance interpretability and alleviate the black-box nature of deep learning, we employ Gradientweighted Class Activation Mapping (Grad-CAM) Selvaraju et al. (2017) to visualize the regions that contribute most to the model’s predictions. Grad-CAM generates class-specific heatmaps by leveraging gradients flowing into the final convolutional layer, highlighting spatially discriminative areas.

**Figure 9:**
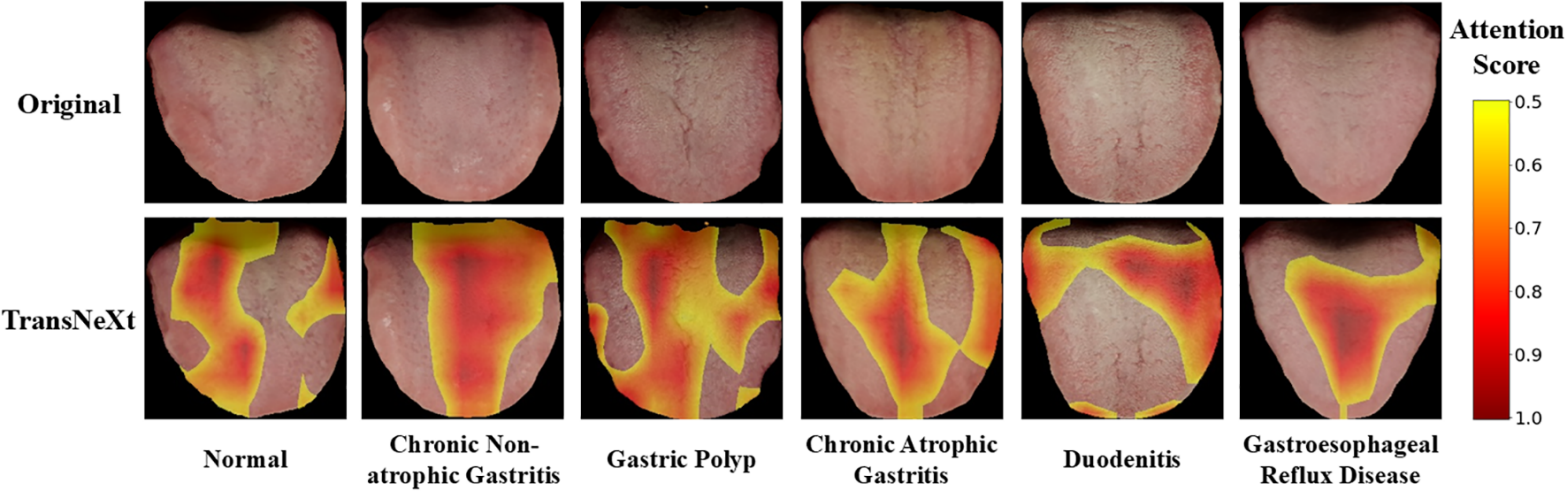
Grad-CAM visualizations for upper gastrointestinal disease classification. Heatmaps highlight the tongue regions most influential for model predictions (red indicates high importance). The model focuses on areas consistent with the TCM principle that the central tongue reflects the spleen and stomach, demonstrating alignment with clinical prior.

As shown in **Figure 9**, for chronic non-atrophic gastritis and gastric polyp, the model’s attention is primarily centrally located. For chronic atrophic gastritis and gastroesophageal reflux disease, the activation is positioned relatively lower within the central zone. For duodenitis, the model focuses on the middleposterior region with a right-side bias. In contrast, for normal samples, the model exhibits diffuse and scattered activation without a concentrated focus. Overall, despite minor variations, the activation for all pathological classes predominantly falls within the central longitudinal zone of the tongue. Notably, the model’s attention to the central tongue region for gastric conditions aligns with the TCM principle that the central tongue reflects the spleen and stomach. This indicates that the proposed method captures clinically meaningful patterns and enhances interpretability.

## 5 Discussion

This study presents a framework for automated tongue image analysis that integrates deep learning-based visual features with handcrafted morphological descriptors. A key insight is the synergistic interaction between feature fusion and data augmentation. Performance gains from augmentation are consistently more pronounced after fusion. This amplification effect suggests that handcrafted features enhance the discriminative structure of the representation space, enabling augmented samples to contribute more effectively to classifier training, while augmentation enriches training diversity to facilitate robust crossmodal feature learning.

### 5.1 Framework advantages

Deploying tongue image analysis in clinical practice entails several practical challenges, including variations in illumination and acquisition devices, tongue protrusion pose, and the inherently complex relationship between tongue manifestations and underlying pathologies. Our framework incorporates multiple design choices to mitigate these issues in practice.

To ensure standardized acquisition, we employ a custom-developed intelligent tongue diagnosis instrument featuring a dark chamber and industrial-grade LED panels. The device provides operators with a real-time visual prompt frame; only images captured with the tongue fully protruding within this frame are accepted, which enforces consistent tongue positioning and reduces pose-related variability. Following acquisition, robust tongue region extraction is performed using SAM-based segmentation with boundary refinement, applied before any augmentations to ensure subsequent transformations operate on anatomically precise regions. We then employ a clinically plausible augmentation strategy that applies random geometric and photometric transformations to simulate variations across imaging conditions and enhance model invariance.

Beyond acquisition and preprocessing, the integration of handcrafted features introduces an inductive bias grounded in established clinical knowledge, which helps reduce the risk of learning spurious correlations from data. The proposed CA-Gate fusion module further leverages handcrafted features as a supervisory prior to guide deep representations, promoting more clinically consistent feature learning under heterogeneous patient conditions.

This design resonates with broader efforts in medical imaging to combine multi-scale features with attention mechanisms. For gastrointestinal disease diagnosis using endoscopic imagery, Khan et al. (2024) proposed a multi-module framework that extracts local and global features via a Local-Global CNN and employs an attention module to focus on critical regions. While their work shares our motivation to integrate multi-scale feature extraction with attention guidance, it differs in two key respects. First, our approach leverages non-invasive tongue images, offering a more accessible screening solution. Second, it does not incorporate handcrafted clinical priors to enhance interpretability, a core design of our CA-Gate fusion module.

From a translational perspective, this framework transforms a subjective, experience-dependent practice into a quantitative tool for gastrointestinal disease screening. As an intelligent pre-screening triage system, it can optimize gastroscopy utilization, reduce unnecessary invasive procedures, and improve resource allocation. These features make it suitable for rapid clinical assessment and community screening. TransNeXt-Tiny achieved substantial improvements over other backbones, with Macro-F1 gains ranging from 0.9% to 5.2% and corresponding sensitivity improvements of 1.0% to 4.8% across different baseline models. These differences were confirmed to be statistically significant through paired t-tests (*p<* 0.05 for all comparisons). In the context of large-scale screening, such gains translate into a meaningful number of additional true positive detections, enabling earlier clinical intervention. Moreover, the integration of handcrafted clinical features not only contributes to the accuracy gain but also enhances interpretability. We further strengthen interpretability by visualizing the model’s attention patterns using Grad-CAM, confirming that the model focuses on clinically relevant tongue regions consistent with TCM knowledge.

### 5.2 Limitations and future directions

Our current framework is designed for single-label classification of five major GID categories. In this study, cases with complex comorbidities were excluded to ensure diagnostic clarity for primary disease classification. Therefore, the model’s performance in patients with multiple concurrent conditions remains to be validated. Extending the framework to multi-label scenarios is an important direction for future work.

Leave-one-center-out cross-validation confirmed robust cross-center generalization across the three clinical centers, indicating that the model captures disease-relevant patterns rather than center-specific artifacts. Nevertheless, broader external validation across more diverse populations and imaging devices remains necessary to fully assess generalizability. Interpretability also remains primarily descriptive, although our fusion mechanism incorporates handcrafted clinical features to enhance transparency, pathophysiology-aligned explanations through correlation with clinical biomarkers are needed to further build clinical trust.

Additionally, we emphasize that our framework is intended as an auxiliary screening tool rather than a standalone diagnostic system. The relationship between tongue manifestations and gastrointestinal diseases is inherently indirect and largely associative rather than strictly causal. Therefore, our model leverages statistical patterns learned from multicenter clinical data to provide supportive evidence for screening, rather than making definitive clinical diagnoses. Future work will focus on integrating clinical biomarkers and longitudinal data to better explore potential causal relationships.

## 6 Conclusion

This study presents a robust computational framework for computer-aided tongue image analysis. By leveraging the TransNeXt hybrid architecture, we capture both local morphological details and global contextual relationships inherent in tongue images, overcoming the limitations of single-type feature extraction networks. Furthermore, the proposed Channel Attention Gated fusion module enables refined integration of heterogeneous modalities through asymmetric recalibration and prior-guided adaptive gating, preserving the supervisory role of clinical knowledge in the fusion process. Validated on a multicenter GID dataset, the framework demonstrates robust discriminative capability and synergistic benefits between multimodal feature learning and data augmentation. These results establish a foundation for transforming traditional tongue diagnosis into an objective, quantitative screening tool with practical clinical utility.

## Data Availability

The original contributions presented in the study are included in the article/**Supplementary Material**. Further inquiries can be directed to the corresponding author.
