# Supplementary material for: Prior-guided feature fusion for tongue image-based gastrointestinal disease auxiliary diagnosis
Source: Front Physiol. 2026 Apr 29;17:1811717. doi: 10.3389/fphys.2026.1811717 (PMC13167410; doi:10.3389/fphys.2026.1811717)
Supplement: Supplementary file 1 [file DataSheet1.pdf]

Supplementary Material

1 HYPERPARAMETER SEARCH SPACE

Table S1. Hyperparameter Search Spaces for Diagnostic Classifiers

Model	Hyperparameter	Search Space	Description
SVM	C	LogUniform(1×10^{-3}, 1×10^{3})	Regularization strength
	gamma	Uniform(1×10^{-4}, 1) (15 values)	Kernel coefficient
	kernel	{linear, rbf, poly}	Decision boundary type
	class_weight	{None, balanced}	Class imbalance handling
LR	C	{0.01, 0.1, 1, 10, 100}	Inverse regularization strength
	class_weight	{None, 'balanced'}	Class imbalance handling
RF	n_estimators	{50, 100, 200}	Number of trees
	max_depth	{None, 10, 20, 30}	Maximum tree depth
	class_weight	{None, 'balanced'}	Class imbalance handling
KNN	n_neighbors	{3, 5, 7, 9}	Number of neighbors
	weights	{uniform, distance}	Weight function for prediction
MLP	hidden_layer_sizes	{(64,), (128,), (256,), (128, 64)}	Neurons per hidden layer
	alpha	{1×10^{-4}, 1×10^{-3}, 1×10^{-2}}	L2 penalty on weights
	learning_rate_init	{1×10^{-3}, 1×10^{-2}}	Initial learning rate
XGBoost	n_estimators	{50, 100, 200}	Number of boosting rounds
	learning_rate	{0.01, 0.1, 0.2, 0.3}	Step size shrinkage
	max_depth	{3, 5, 7}	Maximum tree depth
	subsample	{0.8, 1.0}	Row sampling ratio
	colsample_bytree	{0.8, 1.0}	Feature sampling ratio
	reg_alpha	{0, 0.1, 1}	L1 regularization term
	reg_lambda	{1, 10}	L2 regularization term

2 QUANTITATIVE RESULTS

Table S2. Quantitative Comparison of Different Deep Feature Extraction Models with Data Augmentation

Models	Accuracy	Precision	Recall	Specificity	Macro-F1	AUC	Kappa
Resnet-50	0.810	0.820	0.813	0.816	0.815	0.855	0.772
ConvNeXt-Tiny	0.822	0.830	0.825	0.825	0.826	0.866	0.785
ViT-Base	0.819	0.825	0.822	0.826	0.823	0.864	0.782
Swin-Tiny	0.832	0.833	0.835	0.837	0.833	0.873	0.797
CSWin-Tiny	0.838	0.840	0.843	0.841	0.841	0.877	0.805
BiFormer-Small	0.846	0.851	0.848	0.847	0.849	0.882	0.814
TransNeXt-Tiny	0.853	0.860	0.854	0.855	0.857	0.885	0.819

Table S3. Performance Comparison of Different Downstream Classifiers with Data Augmentation

Classifiers	Accuracy	Precision	Recall	Specificity	Macro-F1	AUC	Kappa
KNN	0.858	0.870	0.862	0.860	0.863	0.887	0.829
RF	0.860	0.863	0.868	0.862	0.864	0.888	0.831
LR	0.863	0.869	0.866	0.865	0.866	0.890	0.835
XGBoost	0.868	0.884	0.872	0.873	0.874	0.894	0.837
MLP	0.870	0.875	0.877	0.874	0.876	0.895	0.838
SVM	0.874	0.881	0.878	0.877	0.879	0.898	0.842

Table S4. Quantitative Comparison of Different Deep Feature Extraction Models without Data Augmentation

Models	Accuracy	Precision	Recall	Specificity	Macro-F1	AUC	Kappa
Resnet-50	0.766	0.788	0.770	0.772	0.774	0.811	0.718
ConvNeXt-Tiny	0.780	0.793	0.784	0.785	0.787	0.825	0.735
ViT-Base	0.778	0.790	0.780	0.778	0.782	0.823	0.733
Swin-Tiny	0.784	0.791	0.787	0.789	0.788	0.832	0.739
CSWin-Tiny	0.790	0.804	0.795	0.794	0.796	0.836	0.747
BiFormer-Small	0.795	0.802	0.799	0.798	0.800	0.839	0.753
TransNeXt-Tiny	0.799	0.806	0.805	0.808	0.805	0.844	0.759

Table S5. Performance Comparison of Different Downstream Classifiers with Data Augmentation

Classifiers	Accuracy	Precision	Recall	Specificity	Macro-F1	AUC	Kappa
KNN	0.801	0.806	0.805	0.806	0.805	0.846	0.760
RF	0.809	0.814	0.812	0.814	0.813	0.852	0.770
LR	0.804	0.805	0.809	0.803	0.808	0.848	0.765
XGBoost	0.812	0.820	0.816	0.818	0.816	0.856	0.774
MLP	0.817	0.821	0.825	0.821	0.822	0.862	0.781
SVM	0.821	0.828	0.825	0.823	0.826	0.866	0.784

Table S6. Performance Comparison of Different Projection Dimensions in MCA

d_z	Accuracy	Precision	Recall	Specificity	Macro-F1	AUC	Kappa
128	0.870	0.868	0.880	0.869	0.871	0.895	0.837
384	0.868	0.888	0.871	0.871	0.874	0.893	0.834
512	0.863	0.871	0.869	0.873	0.869	0.890	0.828
576	0.872	0.884	0.874	0.876	0.877	0.897	0.839
784	0.857	0.866	0.860	0.858	0.864	0.887	0.821
256	0.874	0.881	0.878	0.877	0.879	0.898	0.842
